# Additive Manufacturing-Based In Situ Consolidation of Continuous Carbon Fibre-Reinforced Polycarbonate

**DOI:** 10.3390/ma14092450

**Published:** 2021-05-09

**Authors:** Andreas Borowski, Christian Vogel, Thomas Behnisch, Vinzenz Geske, Maik Gude, Niels Modler

**Affiliations:** Institute of Lightweight Engineering and Polymer Technology, University of Dresden, Holbeinstraße 3, 01307 Dresden, Germany; Christian.Vogel@tu-dresden.de (C.V.); Thomas.Behnisch@tu-dresden.de (T.B.); Vinzenz.Geske@tu-dresden.de (V.G.); Maik.Gude@tu-dresden.de (M.G.); Niels.Modler@tu-dresden.de (N.M.)

**Keywords:** additive manufacturing, in situ consolidation, continuous fibres, composites, flexural properties, porosity

## Abstract

Continuous carbon fibre-reinforced thermoplastic composites have convincing anisotropic properties, which can be used to strengthen structural components in a local, variable and efficient way. In this study, an additive manufacturing (AM) process is introduced to fabricate in situ consolidated continuous fibre-reinforced polycarbonate. Specimens with three different nozzle temperatures were in situ consolidated and tested in a three-point bending test. Computed tomography (CT) is used for a detailed analysis of the local material structure and resulting material porosity, thus the results can be put into context with process parameters. In addition, a highly curved test structure was fabricated that demonstrates the limits of the process and dependent fibre strand folding behaviours. These experimental investigations present the potential and the challenges of additive manufacturing-based in situ consolidated continuous fibre-reinforced polycarbonate.

## 1. Introduction

Unidirectional 3D-printed continuous carbon fibre-reinforced composites have enhanced properties like high tensile strength and high modulus of elasticity along the fibre axis [1]. In addition, composite materials have an excellent stiffness to weight ratio and therefore these materials are mostly used in industries like aviation, aerospace and motor sports. Since Markforged made the continuous fibre-reinforced additive manufacturing technology available in general, the method to produce thermoplastic parts with a local efficient reinforcement in the direction of load, has led to a multitude of scientific investigations in the area of material science, simulation techniques and process optimization [2]. Additive manufacturing and all the advantages associated with it will be an important part of the manufacturing industry in the future. Key advantages are the freedom of design when developing new products, its ability to produce complex parts without the need for moulds and costly subtractive manufacturing methods and the potential to customize products [3]. Especially the Fused Filament Fabrication (FFF) method, which is predestined to add, for instance, reinforcement fibres, fillers and other organic or inorganic particles, opens up the possibility to fabricate parts with tailored properties in small up to midrange quantities. In addition, Reinforced Fused Filament Fabrication (RFFF) with continuous fibres allows for the opportunity to produce parts with enhanced mechanical properties especially in combination with shape and load driven optimized geometries with customized wall thicknesses and infill structures. The consistent application of these advantages results in highly integrated lightweight structures. For this reason, there already are several companies working intensively on developing the technology of thermoplastic continuous fibre-reinforced 3D printing. Anisoprint, for example, has created a co-extrusion process that enables the production of a continuous fibre-reinforced thermoset–thermoplastic composite material (bi-matrix composite) and already offers a particularly high variety of material combinations. The co-extrusion process allows the variation of the fibre volume content of the composite material, which can be controlled within a range of 25–35% [4]. As composites produced using these additive manufacturing exhibit a lower portion of fibres when compared to conventional manufacturing processes such as vacuum resin infusion, thermoforming or prepreg manufacturing with subsequent autoclaving, the mechanical properties are also reduced. Therefore, Arevo and 9T-Labs focus on the production of composite materials with a high fibre volume content and low porosity, whereby post-processes like press moulding, milling and tempering are required to lower porosities and achieve enhanced mechanical properties [5,6]. However, it would be desirable to improve the quality of the in situ produced composite material by increasing the mechanical properties [7]. This could lead to an even higher benefit from a locally as well as layer dependent variable continuous fibre separation strategy, especially for multi material printing systems.

The objective of the investigation described in this paper was to increase the mechanical properties of specimens and components created in an in situ 3D-printing process and to evaluate their improved properties by a standardised test procedure for unidirectional composite materials. In this study, the RFFF method is used to fabricate unidirectional, continuous fibre-reinforced test specimens and a curved geometry to analyse material properties and issues as well as process limits from the developed extrusion based 3D-printing system for pre-impregnated composite materials. Significant research was put into the optimization of process parameters to achieve better mechanical properties [8,9], also the deformation and folding process in curved sections were analysed and described [10]. In the end, an interaction of all mutually influencing parameters is responsible for a sufficient composite quality. The aim of this scientific work was to bring these aspects from the folding behaviour of fibre strands and optimization of process parameters together and evaluate the process boundaries and limits of additive manufacturing-based in situ consolidated composites with an increased fibre volume content. Hereby, the deformation behaviour of the fibre bundles in curved sections and the resulting porosities are of special interest as the possibility to produce components with bent contours distinguishes Automated Tape Laying (ATL) processes from continuous fibre-reinforced 3D-printing.

## 2. Materials and Methods

### 2.1. Equipment and Process Parameters

Three-point bending test specimens were in situ consolidated to evaluate flexural properties in the direction of fibres and a curved structure was fabricated to analyse fibre bundle deformations in areas with high curvature. Therefore, an RFFF-based 3D-printing system (Amplifier) was developed at the Institute of Lightweight Engineering and Polymer Technology (ILK) for the in situ consolidation of pre-impregnated products.

**Figure 1:**
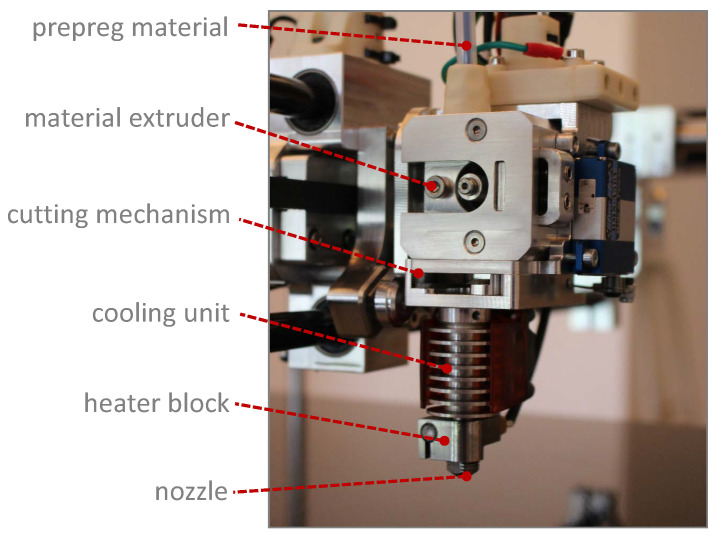
Printhead for the in situ consolidation of continuous fibre-reinforced materials.

The print environment is closed and the heated bed can support temperatures of 140 °C. The nozzle of the printhead to discharge continuous fibre-reinforced materials, shown in Figure 1, allows for temperatures up to 420 °C and is able to melt most relevant technical polymers like Polyamide 6 (PA6), Polycarbonate (PC), Polyetherimide (PEI), Polyetherketoneketone (PEKK) and just falls short of Polyehteretherketone (PEEK). The special feature of the printing system is the variability of semi-finished products, so that a large number of different materials can be processed. The scientific work of Czasny, M. et al. and Domm, M. et al. [8,9] showed that process parameter variation can have a high impact on the resulting material properties. The most relevant parameters used for the investigation are shown in Table 1. The listed print settings were used for all specimens and the curved structure was examined after the process. To determine a nozzle temperature that provides high mechanical properties the temperature was increased by 10 °C, resulting in 3 test series. The first test series was printed with a nozzle temperature of 340 °C, the second with 350 °C and the last with 360 °C.

Flexural properties as well as the folding mechanisms in the bending areas of the print path were especially influenced by the geometrical shape of the cross section. Curved sections are therefore particularly responsible for effects of introduced tensions in the composite part [11].

### 2.2. Material Specification

The used polycarbonate matrix material is Makrolon© and the reinforcement is the carbon fibre Sigrafil© 50K made by SGL. This pre-impregnated material is used in thermoforming processes and should serve as a benchmark for comparison. All relevant properties of a thermoformed test structure are provided in Table 2.

### 2.3. In Situ Consolidation of Carbon Fibre-Reinforced Polycarbonate

**Figure 2:**
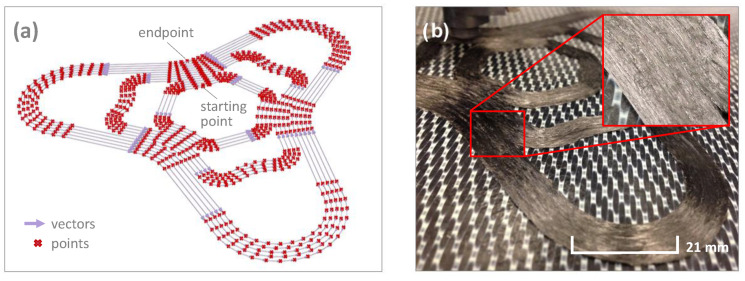
(**a**) Calculated print path (**b**) In situ consolidated carbon fibre-reinforced polycarbonate composite.

A software tool was developed to calculate the machine code to produce the continuous fibre-reinforced test specimens, which enables a curve-based deposition strategy. The print path with its cross-section, layer width and height, is based on two-dimensional vectors with a curvature-dependent point distance. The higher the curvature, the smaller the distance. In this way, a high resolution is achieved in areas of high curvature and areas of low curvature result in a reduced point density. A minimum distance for strong curvatures was defined, so that an unnecessary increase in the number of points is avoided. After providing the machine code, the test structure is in situ consolidated. Figure 2 shows the calculated print path on the left side and the in situ consolidated test structure on the right side.

In situ fabrication refers to the consolidation of continuous carbon fibre-reinforced plastic directly after discharge from the nozzle. This means that the component is completely finished after completion of the last print run, provided that no further post-processing is required. As a rule, a printed component is mostly machined and/or annealed so that surfaces can be refined or mechanical properties can be increased. In these investigations, neither of the surfaces are machined nor are the components subsequently annealed, so that the raw mechanical properties of the in situ consolidation can be analysed.

### 2.4. Standardised Test Method for Bending Properties

**Figure 3:**
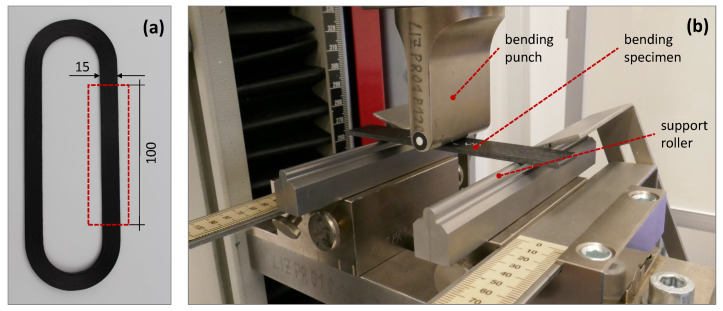
(**a**) Loop structure for two bending test specimens (**b**) Three-point bending test DIN EN ISO 14125 [13].

The bending test is a suitable method to compare process parameters with resulting material properties [9]. The flexural properties that are determined apply exclusively in fibre direction. The bending specimen, which in principle is regarded as and supported like a beam, gets deformed under load at a constant speed until its structural failure. Figure 3 shows the in situ consolidated loop structure and experimental setup for the bending test. Force and deflection are measured to determine the relevant bending properties. This standardised method was selected to achieve the structure characteristics, as these are essential to compare the properties of the thermoformed carbon fibre-reinforced material with those of the in situ consolidated resulting structure. The effort of specimen production is limited to the additive manufacturing process of the unidirectional test specimens according to DIN EN ISO 14125. The test specimens do not require any gluing or doubling, which is usually typical for composites, and can be tested after precise cutting and conditioning. The dimensions correspond to test specimen class IV with a total length of 100 mm, with of 15 mm and thickness of 2 mm [13]. The relevant test conditions are listed in Table 3.

### 2.5. Curvages and Foldings

**Figure 4:**
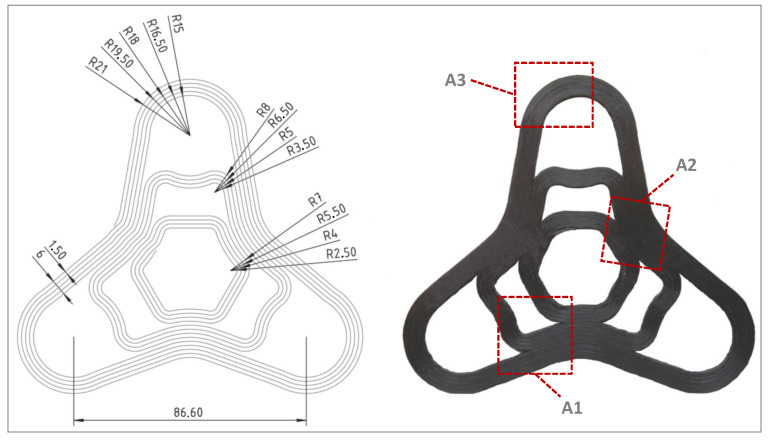
Dimensions of test geometry and in situ consolidated curved test structure with marked areas of interest A1, A2 and A3.

A test geometry was designed, presented in Figure 4, containing different radii and curvature ranges. Component areas without a high curvature should not experience large fibre bundle deformations, but component areas with a high curvature should clearly produce this folding behaviour of the fibre bundles. The phenomena that occur in highly curved areas are examined in more detail and described in Section 3.2. In the best, but only theoretical case, there are no gaps between the print paths and there are no folds in the radii. In reality, due to the defined fibre lengths that must be discharged and the varying print path length due to geometric conditions, there are always folds in curvatures. Compression and tension areas are created, whereby the compression areas withstand lower loads than the tension areas. This is due to the material behaviour of continuous fibre composites and must be taken into account in the process and part design.

### 2.6. Material Structure Evaluation

Computed tomography (CT) is used for a detailed structure analysis of the fabricated composite structures [14,15]. With the help of this non-destructive test method, it becomes possible to evaluate the structure characteristic on a microscopic level and to understand the relationship between fibre deposition and inter- and intra-fibre quality. For this purpose, a GE Vtomex L450 was used with the following specifications shown in Table 4.

**Figure 5:**
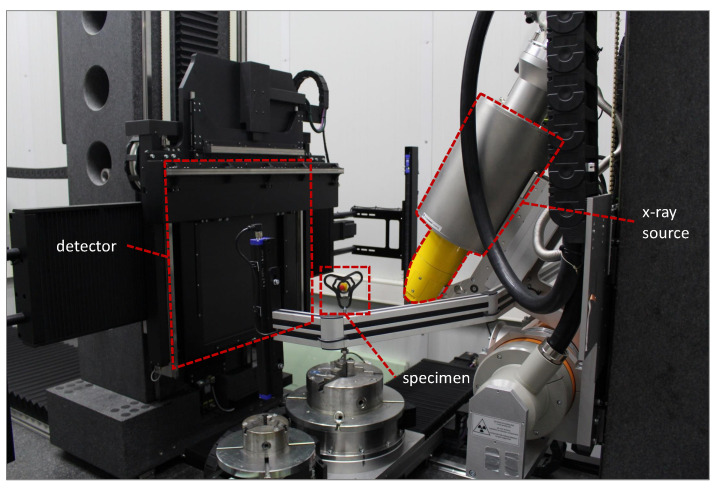
Test setup GE Vtomex L450, curved structure.

Thus, conclusions can be drawn about the composite quality, the pore size and distribution as well as orientation of individual fibres. With this understanding, the parameter set for the printing equipment can also be improved to enhance composite quality. Figure 5 shows the principle test setup for measuring the test structure.

## 3. Results and Discussion

### 3.1. Flexural Strength of CF-PC

**Figure 6:**
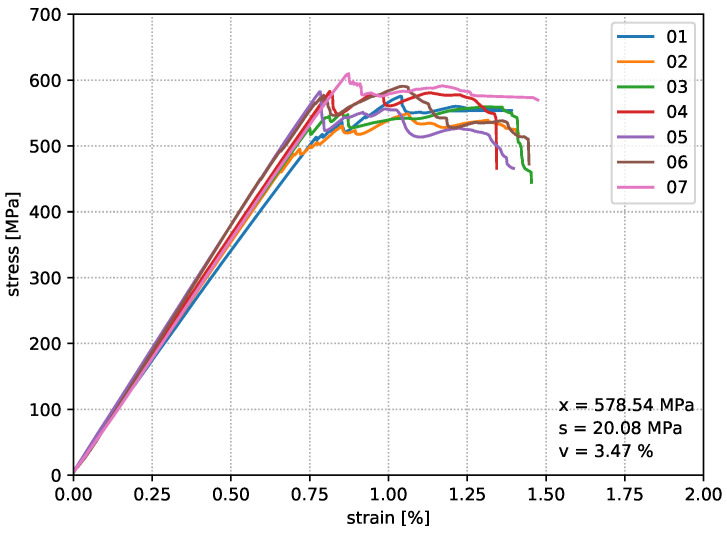
Test results of Series I in situ consolidated with a nozzle temperature of 340 °C.

**Figure 7:**
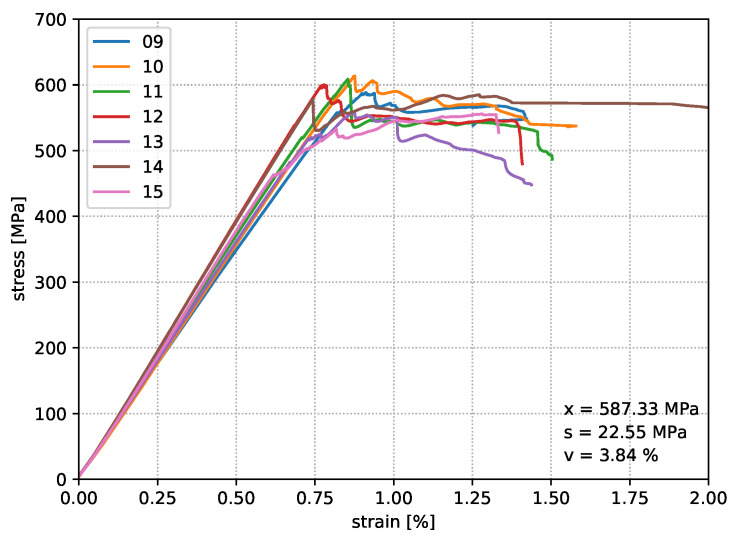
Test results of Series II in situ consolidated with a nozzle temperature of 350 °C.

**Figure 8:**
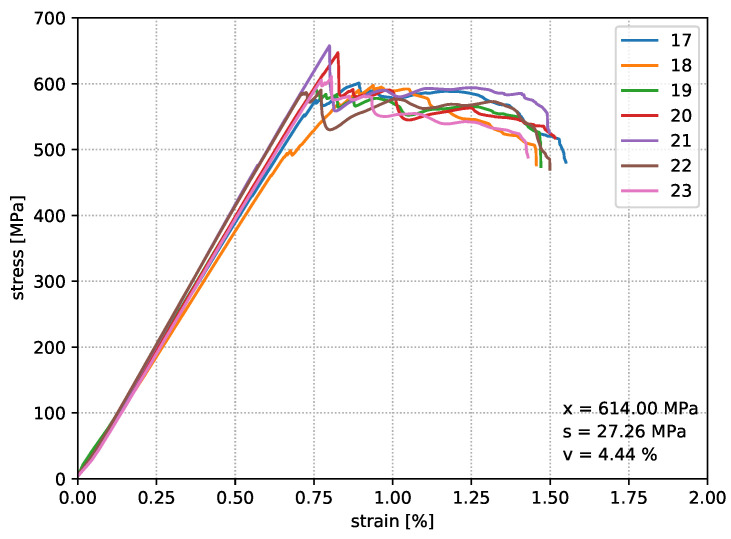
Test results of Series III in situ consolidated with a nozzle temperature of 360 °C.

For the comparability of different nozzle temperatures on the resulting structural properties, eight specimens are produced for each parameter setup, whereby seven are used for the bending tests and one specimen is kept for material analyses. Loop geometries were fabricated according to the model of print path creation from Figure 1, which provides two bending samples per print job. Figure 6, Figure 7 and Figure 8 show the deformation behaviour.

**Figure 9:**
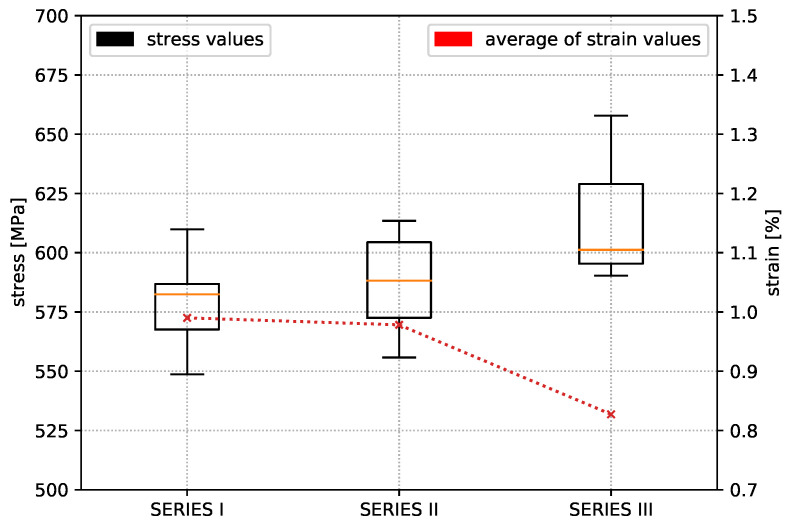
Comparison of test series and corresponding strain values.

The results of test Series I show a repeatable, accurate bending strength around 578 MPa, but the temperature increase by 10 °C in test Series II already reaches an average value of the bending strength around 587 MPa. Increasing the nozzle temperature again leads to a further increase in the characteristic values. Finally, an average value of 614 MPa was determined, which means an improvement of this property by 5.8% compared to test Series I. Figure 9 shows that the strain value of the test Series III drops significantly in comparison to test Series I and II. In the end, it must be stated that the flexural strength of the in situ consolidated composite test specimens corresponds to 87.7% of the flexural properties from the thermoformed unidirectional carbon fibre polycarbonate composite material. All experimentally determined characteristic values from the bending specimens of test Series III are listed in Table 5.

### 3.2. Resulting Folding Effects

**Figure 10:**
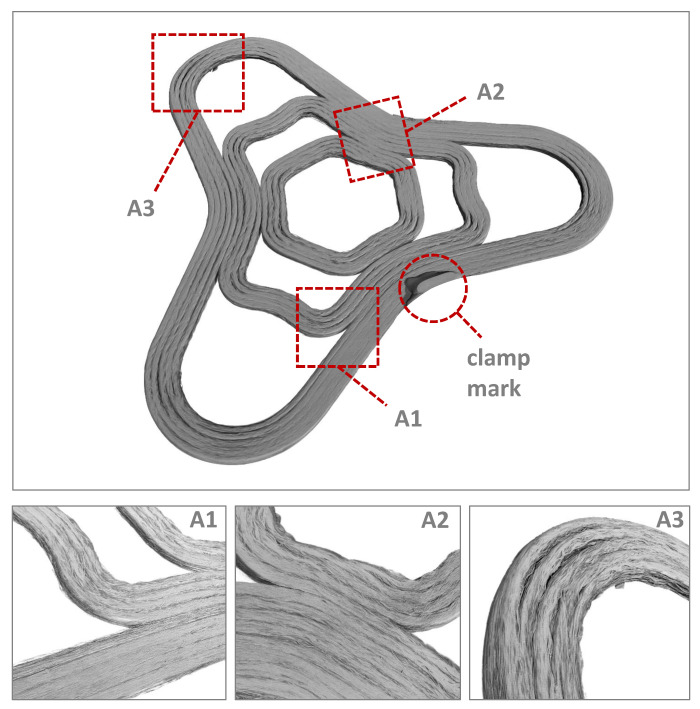
3D reconstruction of the curved structure, process parameters of Series III.

**Figure 11:**
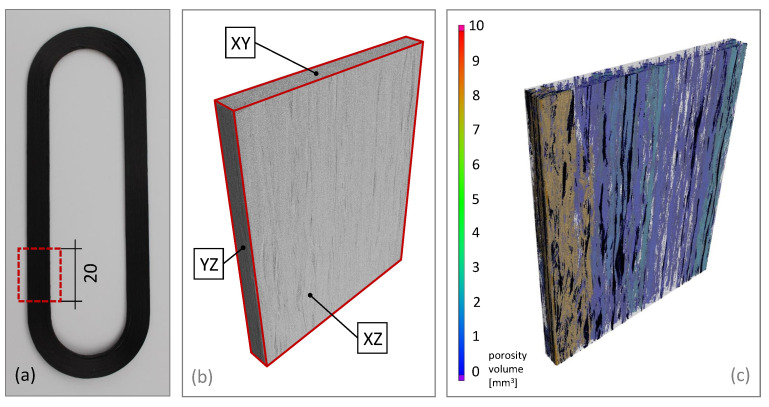
(**a**) Loop structure of Series III and marked area of interest (**b**) 3D reconstruction of the loop section (**c**) Visualisation of calculated porosity.

After the test structure has been in situ consolidated and analysed in the computer tomograph, conclusions can be drawn from the fabrication process to the resulting composite material properties. Figure 10 shows the entire 3D reconstructed curved test geometry and the calculated deposition strategy presented in Figure 2 as well as the characteristics of the deposited filament strands. Due to the unevenness of the printing platform, the filament strands of the first layer could not completely fused together. For this reason, the bending specimens were fabricated in an area with particularly little deviation, which led to a significant improvement in strand adhesion, see Figure 11.

Nevertheless, in areas without strong curvature, an almost closed surface was identified near Areas A1 and A2. A smooth and closed surface indicates that the fibre strands are well consolidated with each other. If gaps or small cavities form between the fibre strands, porosities are created in the component, which means a weakening of the component stability under an external load. Pores and voids are also the starting point for cracks in composite materials. For a complete consolidation of the fibre strands, a very precise and flat printing platform is absolutely necessary. Active measurement of the printing platform before production with active height compensation would be even more beneficial to achieve the best composite quality.

### 3.3. Resulting Micro Structures

After the flexural strengths of the bending specimens have been experimentally determined, the porosities can be analysed. This is important, as the stress–strain curves obtained from the flexural tests do not describe the complex anisotropic material behaviour of the consolidated composite.

**Figure 12:**
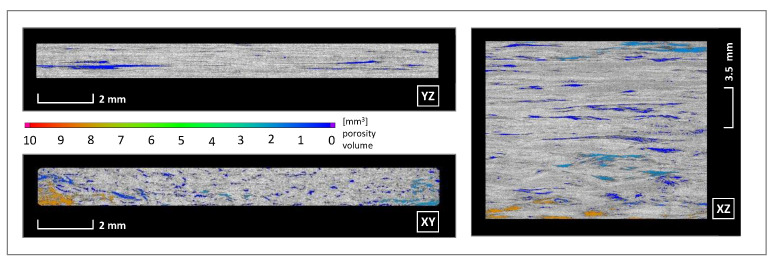
Visualisation of calculated porosity, cross sections XY, XZ and YZ.

Figure 11 shows the 3D reconstruction of a specimen section of test Series III, which achieved the highest bending properties. Note the designation of the sectional planes with reference to Figure 12. The porosity inside the composite material is calculated from the reconstruction data and it is easy to see that the porosity in the left front area (coloured brown) is significantly higher than in the rest of the sample, which may have lacked the necessary compression during consolidation. The high amount of blue coloured voids, with a porosity volume in the range of 0.01 to 1 ${\mathrm{mm}}^{3}$, are distributed throughout the whole sample. This micro porosity due to processing already contained in the pre-impregnated semi-finished product and cannot be avoided during the in situ consolidation. Overall, the in situ consolidated material has a porosity of 6.86%. However, it might be possible to reduce the porosity by applying more compression through the nozzle on the discharged fibre strands. This could be done, for example, by slightly reducing the layer height.

Figure 12 shows sectional cuts from a specimen so that the porosity can be shown per plane. The small gaps between the print paths follow the print direction and illustrate a lack of parallelism depositing process. The waviness in the XZ plane, which is caused by pressing and pulling on the fibre material during the forming process becomes obvious. In sum, all these material defects and the rapid consolidation explain the lower bending strength compared to the thermoformed composite material provided in Table 2.

## 4. Summary and Conclusions

In this study, the Reinforced Fused Filament Fabrication (RFFF) method was used to in situ consolidate unidirectional, continuous fibre-reinforced test specimens. It was shown that the flexural strength of a 3D-printed in situ continuous fibre-reinforced composite material can be enhanced by an optimization of the process parameters. Flexural properties were determined with a three-point bending test and in addition to these test results, porosity was also taken into account for the evaluation. A comparison of flexural strength for in situ consolidated and thermoformed unidirectional carbon fibre-reinforced polycarbonate as a benchmark was conducted. The flexural strength of the in situ consolidated composite test specimens corresponds to 87.7% of the flexural properties from the thermoformed composite material without any post-processing. The porosity is 6.86% and can likely be reduced by additional compression on the fibre strands directly after discharge from the nozzle through a slight reduction of the print path height. Furthermore, a highly curved test structure was produced, which enabled an evaluation of the fibre folding behaviour. It has been found that even highly curved areas can be reliably consolidated, but insufficient strand adhesion has to be expected here. In order to increase component performance, it is necessary to evaluate process limitations, such as minimum radii, as these must be taken into account in the design process to ensure efficient material utilisation. In conclusion, these investigations are particularly interesting for multi material additive manufacturing-based continuous fibre-reinforced processes that do not require any post-processing. The development and initial investigations in the current study provide the basis for more in-depth analyses of the relationship between the process parameters and the material microstructure characteristics, respectively mechanical properties. In particular, the influence of curvature level on fibre orientation and the occurring porosity, always regarding the resulting anisotropic material behaviour, is to be clarified more precisely in future studies. Only with this understanding, truly efficient and highly integrated structures can be created.

## Figures and Tables

**Table 1:** Process parameters Amplifier.

Property	Unit	Abbreviation	Value
bed temperature	[°C]	t_b_	100
nozzle temp. Series I	[°C]	t_n1_	340
nozzle temp. Series II	[°C]	t_n2_	350
nozzle temp. Series III	[°C]	t_n3_	360
nozzle diameter	[mm]	d_n_	1.2
print velocity	[mm/s]	v_p_	5.8
humidity	[%]	h	40
path with	[mm]	p_w_	1.5
path height	[mm]	p_h_	0.4

**Table 2:** Properties of unidirectional carbon fibre polycarbonate, *Maezio^TM^* CF GP 1000T [12].

Property	Unit	Abbreviation	Value
tensile modulus 0°	[GPa]	E_0°_	105
flexural modulus 0°	[GPa]	E_f_	93
shear modulus ±45°	[GPa]	G	2.2
tensile strength 0°	[MPa]	σ_0°_	1400
flexural strength 0°	[MPa]	σ_f_	700
shear strength ±45°	[MPa]	σ_τ_	40
fibre volume content	[%]	φ	44

**Table 3:** Test requirements Zwick Z2,5 and test conditions.

Parameter	Unit	Value
pre-force	[N]	2
test speed	[mm/min]	2
start e-module determination	[%]	0.05
end e-module determination	[%]	0.25
support range	[mm]	80
radius of support rollers	[mm]	2
radius of the bending punch	[mm]	5
room temperature	[°C]	23
humidity	[%]	50

**Table 4:** Technical characteristics, GE Vtomex L450.

Parameter	Unit	Characteristic
acceleration voltage	[kV]	30–300
X-ray current	[A]	3
target material		tungsten
active detector area	[mm]	400 × 400
detector resolution	pixels	2024 × 2024
greyscale	bit	16

**Table 5:** Properties of Series III, in situ consolidated CF-PC composite.

Property	Unit	Abbreviation	Value
flexural modulus 0°	[GPa]	E_f_	78.9
flexural strain	[%]	ϵ_f_	0.83
flexural strength 0°	[MPa]	σ_f_	614

## Data Availability

Data is contained within the article.

## Abbreviations

The following abbreviations are used in this manuscript:

AM	additive manufacturing
CT	computed tomography
FFF	Fused Filament Fabrication
RFFF	Reinforced Fused Filament Fabrication
ATL	automated tape laying
ILK	Institute of Lightweight Engineering and Polymer Technology
